# The long non-coding RNA *OTX2-AS1* promotes tumor growth and predicts response to BCL-2 inhibition in medulloblastoma

**DOI:** 10.1007/s11060-023-04508-y

**Published:** 2023-11-17

**Authors:** Nan Qin, Eunice Paisana, Daniel Picard, Gabriel Leprivier, Maike Langini, Carlos Custódia, Rita Cascão, Catleen Conrad, Mirko Peitzsch, Anja Stefanski, Kai Stühler, Ute Fischer, Claudia C. Faria, Sascha Dietrich, Guido Reifenberger, Marc Remke

**Affiliations:** 1 Department of Hematology, Oncology, and Clinical Immunology, Medical Faculty, Heinrich Heine University, University Hospital Düsseldorf, Düsseldorf, Germany; 2 Department of Pediatric Oncology, Hematology, and Clinical Immunology, Medical Faculty, Heinrich Heine University, University Hospital Düsseldorf, Düsseldorf, Germany; 3 Institute of Neuropathology, Medical Faculty, Heinrich Heine University, and University Hospital Düsseldorf, Düsseldorf, Germany; 4 High-Throughput Drug Screening Core Facility, Medical Faculty, Heinrich Heine University, Düsseldorf, Germany; 5 Mildred Scheel School of Oncology Aachen Bonn Cologne Düsseldorf (MSSO ABCD), Düsseldorf, Germany; 6 Instituto de Medicina Molecular João Lobo Antunes (iMM), Faculdade de Medicina da Universidade de Lisboa, Lisbon, 1649-028 Portugal; 7 Institute of Clinical Chemistry and Laboratory Medicine, Medical Faculty Carl Gustav Carus, University Hospital Carl Gustav Carus, Technische Universität Dresden, Dresden, Germany; 8 Molecular Proteomics Laboratory, Biological and Medical Research Center (BMFZ), Heinrich Heine University Düsseldorf, Düsseldorf, Germany; 9 Institute for Molecular Medicine 1, Heinrich Heine University Medical Faculty, Düsseldorf, Germany; 10 Department of Neurosurgery, Hospital Santa Maria, Centro Hospitalar Universitário Lisboa Norte, EPE, Lisbon, 1649-028 Portugal; 11 German Cancer Consortium (DKTK), partner site Essen/Düsseldorf, Düsseldorf, Germany; 12 Department of Pediatric Hematology and Oncology, University Medical Center of Saarland, Homburg/Saar, Germany

**Keywords:** Medulloblastoma, LncRNA, OTX2-AS1, BCL-2 inhibitor, High-throughput drug screening

## Abstract

**Purpose:**

Primary brain tumors are a leading cause of cancer-related death in children, and medulloblastoma is the most common malignant pediatric brain tumor. The current molecular characterization of medulloblastoma is mainly based on protein-coding genes, while little is known about the involvement of long non-coding RNAs (lncRNAs). This study aimed to elucidate the role of the lncRNA *OTX2-AS1* in medulloblastoma.

**Methods:**

Analyses of DNA copy number alterations, methylation profiles, and gene expression data were used to characterize molecular alterations of *OTX2-AS1* in medulloblastoma tissue samples. In vitro analyses of medulloblastoma cell models and orthotopic in vivo experiments were carried out for functional characterization of *OTX2-AS1*. High-throughput drug screening was employed to identify pharmacological inhibitors, while proteomics and metabolomics analyses were performed to address potential mechanisms of drug action.

**Results:**

We detected amplification and consecutive overexpression of *OTX2* and *OTX2-AS1* in a subset of medulloblastomas. In addition, *OTX2-AS1* promoter methylation was linked to *OTX2-AS1* expression. *OTX2-AS1* knockout reduced medulloblastoma cell viability and cell migration in vitro and prolonged survival in the D283 orthotopic medulloblastoma mouse xenograft model. Pharmacological inhibition of BCL-2 suppressed the growth of *OTX2-AS1* overexpressing medulloblastoma cells in vitro.

**Conclusions:**

Our study revealed a pro-tumorigenic role of *OTX2-AS1* in medulloblastoma and identified BCL-2 inhibition as a potential therapeutic approach to target *OTX2-AS1* overexpressing medulloblastoma cells.

**Supplementary Information:**

The online version contains supplementary material available at 10.1007/s11060-023-04508-y.

## Introduction

Brain tumors are the most frequent solid malignancies in childhood and one of the leading causes of cancer-related death in this age group. Medulloblastoma is the most common pediatric malignant brain tumor that originates in the cerebellum and can potentially metastasize via the spinal fluid to other parts of the brain and the spinal cord [1]. Long-term survival rates approach 75% in community-based studies, but the long-term consequences of survivorship following treatment are often severely debilitating [2, 3]. As a consequence of the disease itself but also the required multimodal aggressive therapy, many, if not most, medulloblastoma survivors are left with long-term physical, neurological, cognitive, reproductive, endocrinological, or psychosocial deficits [4]. Thus, the disease and its treatment represent a significant burden of suffering for the patients and their families and imply a long-term burden on the health care system with a substantial and chronic impact on health care costs [5]. Therefore, there is an urgent need to identify novel targeted therapeutic approaches that are both effective and less toxic.

Based on gene expression and DNA methylome profiles, medulloblastomas are nowadays classified into four molecular groups, namely Wingless (WNT), Sonic Hedgehog (SHH), Group 3 (G3), and Group 4 (G4), with each group being associated with specific genetic alterations and activated signaling pathways [6, 7]. Additional epigenetic and proteogenomic analyses have demonstrated extensive intertumoral heterogeneity, leading to the characterization of molecular subgroups within the SHH and G3/G4 medulloblastoma groups [8, 9]. However, these studies were mainly based on the analysis of protein-coding genes, while the role of non-coding RNAs, including lncRNAs, in medulloblastoma pathogenesis remained elusive. LncRNAs are RNA transcripts longer than 200 base pairs that do not code for proteins [10]. While it is estimated that the human genome contains 58,000 lncRNAs [11], molecular and cellular functions have been identified for only a few of these. LncRNAs are emerging as important new players in cancer biology as they may have regulatory roles in oncogenic and tumor-suppressive pathways [12, 13]. For example, lncRNAs reprogram gene expression by modulating chromatin or directly influencing the transcription machinery, help to diversify the transcriptome and proteome through regulating mRNA processing, and stabilize or destabilize protein complexes by binding or acting as scaffolds for higher-order protein-protein and RNA-protein complexes [14]. More interestingly, about 40% of lncRNAs are expressed in the brain, and their aberrant expression has been linked to neuro-oncological disorders [15]. Notably, recent studies have indicated that lncRNAs are aberrantly regulated in medulloblastoma, where they can function as tumor suppressors or oncogenes. For instance, *NKX2-2AS* has been shown to suppress tumorigenesis in SHH-driven medulloblastoma [16], while *HHIP-AS1*, *TP73-AS1*, *CCAT1*, *CRNDE*, *LOXL1-AS1*, *ANRIL*, and *linc-NeD125* have been reported to promote medulloblastoma cell proliferation and migration [17–23]. Thus, deciphering the roles of lncRNAs in medulloblastoma tumorigenesis may lead to relevant novel insights regarding tumor biology and may eventually inform therapeutic approaches for molecularly targeted treatment of children with this highly aggressive brain tumor.

## Materials and methods

### Cell culture, viral production, and transduction

Cell lines were cultured following Good Cell Culture Practice. Supplementary Table S1 lists the sources of the medulloblastoma cell lines used in this study and the antibiotic-free culture media used for their in vitro cultivation. All cell lines were maintained in a humidified 5% CO2 incubator at a temperature of 37 °C.

Lentivirus was produced by transfecting HEK293T with packaging vectors (pMDLg/pRRE, Addgene#12251; pRSV-Rev, Addgene#12253; pMD2.G, Addgene#12259). Pure populations of the stable cell lines were selected using antibiotics (InvivoGen, Toulouse, France). The concentrations used for the antibiotics treatment of each cell line are listed in supplementary Table S2.

All experiments were performed using authenticated cell lines. Authentication was performed frequently using the Short Tandem Repeat analysis by the Genomics & Transcriptomic Laboratory of the Biological and Medical Research Center (BMFZ) at Heinrich Heine University Düsseldorf, Germany (https://tsm.gtl.hhu.de).

### DNA copy number profiling

DNA copy number profiles were extracted from publicly available data [9]. This dataset contained 1087 unique medulloblastoma cases. Copy number segmentation was performed from genome-wide SNP6 array data using the conumee package (v0.99.4) in the R statistical environment (v3.2.3) [24]. The segmented copy number was used to identify broad copy number events. The log2 R ratio (LRR) of each chromosome was calculated using a size-weighted mean of all segments mapping to the chromosome. If the LRR was greater than 0.2, it was declared gained; if it was less than − 0.2, it was declared lost; and if it fell within the range of -0.2 to 0.2, it was considered balanced. The samples with LRR values greater than 0.2 were used to generate the figure.

### DNA methylation data analysis

Global DNA methylation data were extracted from the published DNA methylation data [6], which analyzed 763 primary samples using Illumina Infinium HumanMethylation450 BeadChips. The obtained data was normalized using the SWAN method, part of the minfi package [25]. After generating beta and logitB values, the NMF package (v0.20.6) was utilized to select the top 10,000 probes displaying the most variable methylation based on the standard deviation. The selected probes were imported into Partek Genomic Suite (Partek Incorporated, St. Louis, USA).

### Identification of *OTX2-AS1* transcriptional isoforms in medulloblastoma

To identify the primary transcriptional isoforms of *OTX2-AS1*, the published RNAseq data from 41 flash-frozen primary medulloblastomas [26] were reanalyzed. We mapped sequencing reads to the transcriptome of Ensembl (www.ensembl.org) and established a baseline for determining the exons by using the mean of noise from 41 samples.

### RNA extraction, reverse transcription and qRT-PCR

RNA was extracted from cell lines using the SV total RNA isolation system (Promega, Mannheim, Germany). Extracted RNA (1–3 µg) according to the manufacturer’s recommendations. RNA was reverse transcribed with M-MLV reverse transcriptase (Promega). The resulting cDNA was subjected to amplification by quantitative reverse transcription PCR (qRT-PCR) using the Promega GoTaq qPCR master mix (Promega). The qRT-PCR analysis was performed with the CFX384 Touch Real-Time PCR detection system (Bio-Rad, Munich, Germany). To reduce the risk of false positives caused by the amplification of any contaminating genomic DNA, PCR primers were designed to span exon-exon junctions. For accurate interpretation of qRT-PCR data, all primers were tested using the primer-template binding efficiency. Primer sequences and their binding efficiency are shown in Supplementary Table S3.

### Recombinant lentiviral vector construction for stable overexpression (OE) of *OTX2-AS1*

To confer stable OE of *OTX2-AS1*, the plasmid LeGO-lnc (a gift from Jan-Henning Klusmann, Addgene plasmid #80624) [27] was used to construct the recombinant lentiviral vector. The plasmid for *OTX2-AS1* OE was generated by subcloning the PCR-amplified *OTX2-AS1* gBlocks (IDT, Leuven, Belgium) fragment into the XbaI and XhoI (NEB Frankfurt am Main, Germany) sites of LeGO-lnc.

### CRISPR/Cas9-mediated knockdown (KD) and knockout (KO) of *OTX2-AS1*

The pLVhU6-sgRNA hUbc-dCas9-KRAB-T2a-Puro plasmid (a gift from Charles Gersbach) [28] was used for the promoter repressor experiment. The sgRNAs (Supplementary Table S4) were designed using the CRISPR design service engine (http://crispr.mit.edu/ and http://sam.genome-engineering.org/). A donor plasmid, OTX2-AS1-Exon1-Puromycin-pUC19, in which a puromycin selection marker and poly (A) tall were flanked by 5´and 3´homology arms, was used as a homology-directed repair template to achieve genomic knock-in. Primers for plasmid cloning and sgRNAs are listed in Supplementary Table S4. The sequences of all expression vectors were confirmed by DNA sequencing and restriction enzyme analysis (data not shown).

### Cell migration assay using the xCELLigence system

Cell migration was monitored in real-time using the xCELLigence system (ACEA Biosciences, San Diego, USA). 2 × 10^4^ cells were seeded in a CIM-Plate 16 in triplicate, and migration behavior was monitored for 72 h. Readings were recorded at 15-minute intervals. Cells maintained in a serum-free medium served as a control, and all other cell index (CI) values were normalized to this baseline.

### Establishment of medulloblastoma xenografts

Six- to eight-week-old NOD.Cg-*Prkdc*^*scid*^*Il2rg*^*tm1Wjl*^/SzJ (NSG) mice were purchased from Charles River Laboratories France (Lyon, France). In accordance with Directive 2010/63/EU (transposed to Portuguese legislation through Decreto-Lei No. 113/2013, of August 7th), all animal procedures were approved by the institutional animal welfare body (ORBEA-iMM). Human endpoints were established for 10% body weight loss, paralysis, and neurological impairment. Six mice were each injected with 250,000 medulloblastoma cells, either expressing *OTX2-AS1* or with *OTX2-AS1* KO, in the right cerebellar hemisphere. All mice were monitored for body weight, discomfort, and distress every other day. Once any of the aforementioned humane endpoints were reached, the mice were euthanized using an anesthetic overdose of sodium pentobarbital.

### CellTiter-Glo luminescent cell viability assay

The CellTiter-Glo reagent (Promega, Madison, USA) was prepared according to the manufacturer’s instructions for the cell viability readout. To ensure logarithmic growth during the 72-hour experiment duration, the cell concentrations used were determined experimentally. Cells were seeded in 384-well plates in 30 µl/well. After incubation, CellTiter-Glo was used to quench cells, and luminescence was measured using a Spark 10 M microplate reader (Tecan, Männedorf, Switzerland).

### Inhibitor libraries and drug screening

Drug screening was performed at the High-throughput Drug Screening Core Facility of the Medical Faculty at Heinrich Heine University Düsseldorf. Sample preparation and data processing were performed as described [29]. An extended library comprising 579 anti-cancer compounds was used for profiling the drug response of medulloblastoma cells in relation to *OTX2-AS1* expression. All compounds were used at concentrations ranging from 0 to 10 µM, covering 6 to 8 different concentration levels. Cellular responses to compounds were based on a normalized area under the dose-response curve (AUC).

### Sample preparation for LC/MS analysis

The cell pellets were dissolved in 50 µl of lysis buffer containing 50 mM Tris, 150 mM NaCl, 0.1% (w/v) SDS, 0.5% (w/v) sodium deoxycholate, 1% (v/v) Triton X-100, Protease Inhibitor Cocktail, 1 mM PMSF, 10 mM sodium azide, 10 mM sodium ascorbate, and 5 mM Trolox. Then, the solution was sonicated on ice. A volume of 12.5 µL per sample was desalted through electrophoretic migration at 50 V for 10 min on a 4–12% Bis-Tris polyacrylamide gel (Novex NuPAGE, Thermo Scientific, Darmstadt, Germany). After silver staining, protein bands were cut out, reduced, alkylated, and digested with trypsin before peptide extraction via sonication. The peptides were dissolved and diluted with 0.1% TFA (v/v).

### Proteomic analysis

For mass spectrometric analysis, 15 µL of peptide solution per sample was analyzed on a nano-high-performance liquid chromatography-electrospray ionization mass spectrometer. Peptides were.

separated at a constant flowrate of 300 nL/min over a 120 min gradient in an analytical column (Acclaim PepMap RSLC C18, 25 cm x 75 μm x 2 μm particle size, 100 Å pore size, Thermo Fisher Scientific) at 60 °C. Separation was achieved through a gradient from 4 to 40% solvent B in solvent A (solvent A: 0.1% (v/v) formic acid in water, solvent B: 0.1% (v/v) formic acid, 84% (v/v) acetonitrile in water). Peptides were ionized at a voltage of 1,400 V and introduced into the mass spectrometer operated in positive mode. MS scans were recorded in profile mode in a range from 350 to 2000 m/z at a resolution of 70,000, while tandem mass spectra were recorded at a resolution of 17,500. Tandem mass spectra were recorded with a data-dependent Top10 method and 30% normalized collision energy. Dynamic exclusion was activated with a repeat count of 1 for 100 s.

### Measurement of fumarate and succinate

For ultrahigh-pressure liquid chromatography tandem mass spectrometry (UPLC-MS/MS) measurements, as described previously [30], cell pellets were extracted following the previously described procedure. Briefly, 1 million cells were washed with PBS, followed by extraction in 500 µl ice-cold LC/MS grade methanol. The homogenates were centrifuged at 2000 g for 5 min at 4 °C, followed by a dry-down of supernatants using a speed vac concentrator (Thermo Fischer Scientific). The residues were then resuspended in a 0.2% formic acid solution, which was cleared with a 0.2 μm centrifugal filter and then directly analyzed by UPLC-MS/MS.

### Computational mass spectrometric data analysis

For peptide/protein identification, Proteome Discoverer (version 2.1.0.81, Thermo Fisher Scientific) utilized Mascot and MS Amanda search engines, both of which employed the UniProt database (human; including isoforms; date 2018-09-12). To ensure accurate identification, a false discovery rate of 1% (p ≤ 0.01) at the peptide level was set. Progenesis QI for Proteomics (Version 2.0, Nonlinear Dynamics, Waters Corporation, Newcastle upon Tyne, UK) was used to quantify proteins. The Bonferroni test ANOVA was used to identify proteins that showed significant expression differences between the two sets of cells.

### Statistical analysis

Cell index for real-time dynamic migration measurement and slope were calculated automatically by the RTCA Software Package 1.2. Normalizations were performed using the RTCA Software Package 1.2. Dose-response curves were generated using non-linear regression in Python (log(concentration of inhibitor) versus response) to evaluate drug response. For normalization, the cell viability at the lowest drug concentration was set to 100%. Biostatistics data were analyzed using Prism 8 software.

## Results

### *OTX2-AS1* is co-amplified with *OTX2* and overexpressed in primary medulloblastoma

**Fig. 1:**
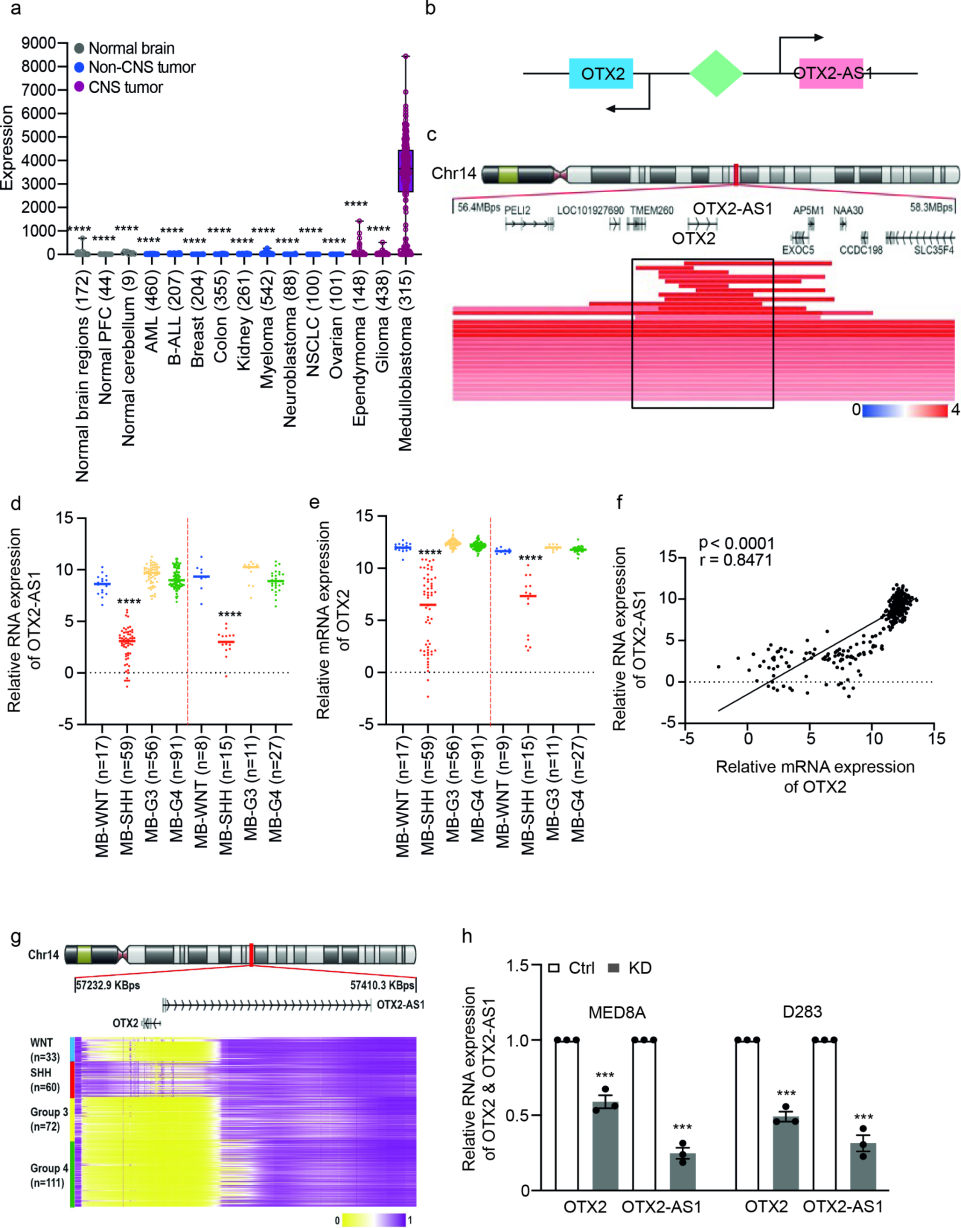
Identification of alteration of*OTX2-AS1* in medulloblastoma. **(a)** MegaSampler analyzes (R2- http://hgserver1.amc.nl) showing expression of *OTX2* (probes 242128_at) in various tissues and tumor entities. **(b)** Illustration of the genomic region on chromosome 14 harboring the OTX2 and OTX2-AS1 loci (red highlighted area) showing co-amplification of *OTX2* and *OTX2-AS1* relative to surrounding genes 500 kb up and downstream of the *OTX2* and *OTX2-AS1* locus in medulloblastoma samples. The black square highlights the minimal overlapping region encompassing focal DNA copy number gains or amplifications centered around *OTX2* and *OTX2-AS1*. **(c, d)** MegaSampler analyses (R2- http://hgserver1.amc.nl) based on published data from two independent studies by Robison et al. [32] and Kool et al. [55], which showed expression of *OTX2-AS1* (probes 1561319_at) **(c)** and *OTX2* (probes 242128_at) **(d)** in medulloblastoma tissues stratified according to molecular group. The p-values were determined by 2-way ANOVA. * Indicates statistical significance between SHH medulloblastoma versus the other medulloblastoma groups. **(e)** *OTX2-AS1* RNA levels are positively correlated with *OTX2* mRNA levels in medulloblastomas. **(f)** Heatmap showing the subgroup-specific methylation patterns of the *OTX2* and *OTX2-AS1* promoter based on data from 763 medulloblastoma samples. The heatmap displays methylation beta values (yellow indicates hypomethylation; pink indicates hypermethylation). **(g)** Relative expression of *OTX2-AS1* and *OTX2* expression in CRISPR/Cas-mediated stable knockdown in D283 and MED8A medulloblastoma cells. The p-values were determined by paired t-test. Values of *p* < 0.05 were considered statistically significant. *** denotes *p* < 0.001

Orthodenticle Homeobox 2 (*OTX2)* is strongly expressed in medulloblastoma compared to normal brain and other tumor entities (Fig. 1a). Transcription of the long non-coding RNA *OTX2-AS1* is driven from the proximal *OTX2* promoter in the opposite direction of transcription factor *OTX2* [31]. To determine genetic alterations and expression of *OTX2-AS1* in medulloblastoma, we comprehensively analyzed DNA copy number alterations targeting the *OTX2-AS1* gene locus and *OTX2-AS1* expression levels in published data sets [9, 32]. First, we focused on the gene locus of *OTX2-AS1* and defined a minimally overlapping region involving a consistent co-amplification of *OTX2* and *OTX2-AS1* (Fig. 1b). Secondly, we interrogated patient-derived data using the R2 platform (www.r2.amc.nl) and confirmed that *OTX2-AS1* expression is significantly upregulated in WNT, G3, and G4 medulloblastoma compared to SHH medulloblastoma (*p* < 0.0001, Fig. 1c). We also observed a similar expression pattern for the adjacent gene *OTX2* gene (Fig. 1d) and significant positive correlation between *OTX2* and *OTX2-AS1* expression (Fig. 1e). By exploring DNA methylation data from tumor tissues [6], we found increased methylation of the promoter of *OTX2*/*OTX2-AS1 in* SHH medulloblastoma compared to WNT, G3 and G4 medulloblastomas, which may contribute to the low expression of these two genes in the SHH group (Fig. 1f). In addition, we designed specific sgRNAs targeting the potential promoter region of *OTX2-AS1* and achieved simultaneous knockdown of *OTX2-AS1* and *OTX2* in medulloblastoma cells (MED8A and D283, Fig. 1g). These observations confirm that *OTX2-AS1* and *OTX2* share a common bidirectional promoter.

### *OTX2-AS1* promotes medulloblastoma cell viability and migration

*OTX2-AS1* is a multiexon lncRNA that gives rise to different alternatively spliced isoforms in human cells (www.ensembl.org). Analysis of published RNAseq data [26] revealed that only *OTX2-AS1-205* is present in medulloblastoma (Fig. S1a).

**Fig. 2:**
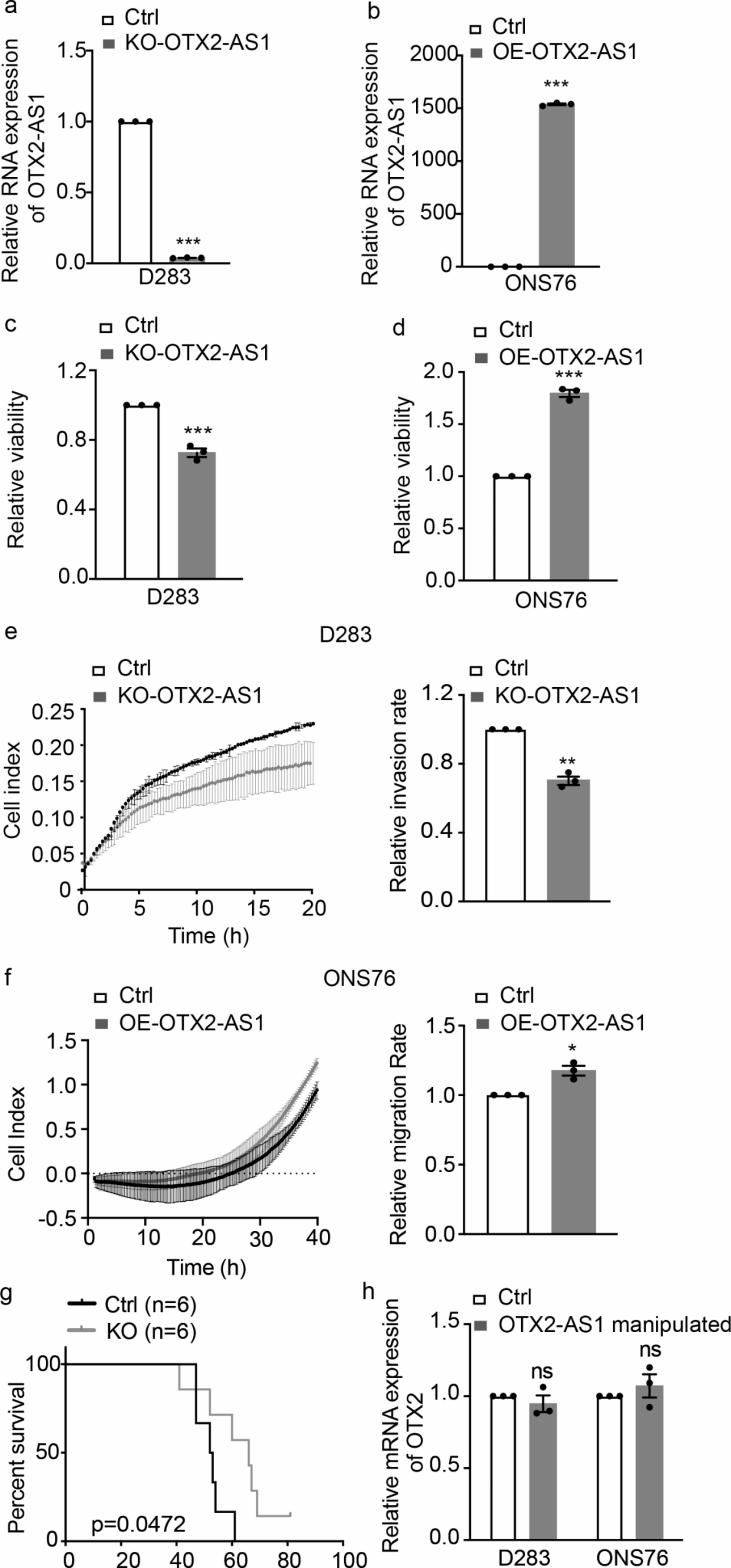
Aberrant *OTX2-AS1* expression promotes aggressive tumor biology in medulloblastoma. **(a, b)** Measurement of stable knockout (KO) of *OTX2-AS1* in D283 medulloblastoma cells **(a)** and stable overexpression (OE) in ONS76 medulloblastoma cells **(b)** using qRT-PCR. *GAPDH* and *PGK1* were used as housekeeping controls. **(c, d)** Measurement of cell viability in D283 *OTX2-AS1* KO **(c)**, ONS76 *OTX2-AS1* OE **(d)**, and their corresponding control cells using the CellTiter-Glo assay. **(e, f)** Measurement of cell migration in D283 *OTX2-AS1* KO **(e)**, ONS76 *OTX2-AS1* OE **(f)**, and their corresponding control cells using the xCelligen live cell migration assay. Experiments were repeated in triplicate; data show the mean ± SEM. The p-values were determined by unpaired t-test. Values of *p* < 0.05 were considered statistically significant. *, **, and *** denote *p* < 0.05, *p* < 0.01, and *p* < 0.001, respectively. **(g)** Kaplan-Meier survival curves for orthotopic mice models (*n* = 6 mice/group) injected with control (Ctrl) or KO-OTX2-AS D283 medulloblastoma cells. The p-value was determined by the log-rank test. **(h)** Measurement of *OTX2* mRNA expression after *OTX2-AS1* KO in D283 cells or *OTX2-AS1* OE in ONS76 cells using qRT-PCR. Experiments were repeated in triplicate; data show the mean ± SEM. The p-values were determined by unpaired t-test. Values of *p* < 0.05 were considered statistically significant. ns means non-significant

To confirm the expression of *OTX2-AS1-205*, we measured the expression of *OTX2-AS1-205* in 10 different medulloblastoma cells (Fig. S1b). Based on the average expression level, most G3 and G4 medulloblastoma cells (D283, MB002, CHLA-01R, CHLA-01, HD-MB03, and MED8A) are classified as high *OTX2-AS1* expressing cells. On the other hand, all SHH medulloblastoma cells (D341, CHAL-259, DAOY, UW228-2, and ONS76) showed low or non-detectable *OTX2-AS1* expression [33]. Therefore, we performed a KO of *OTX2-AS1* in D283 and overexpressed *OTX2-AS1* in ONS76. Stable KO was achieved with CRISPR/Cas9 nickase and paired sgRNAs. Additionally, we utilized a homology repair donor with the antibiotic-selectable marker cassette, allowing for the selection of positive KO cells in a medium containing puromycin. Subsequently, the successful selection of heterozygous and homozygous KO clones was confirmed using PCR to amplify the cleavage region (Fig. S1c). Insertion of poly(A) tail in the first exon of *OTX2-AS1* terminated transcription and resulted in almost a complete downregulation of *OTX2-AS1* in D283 (Fig. 2a). As a counterpart, stable OE of *OTX2-AS1* in ONS76 (Fig. 2b) was achieved using lentivirus carrying a complete *OTX2-AS1-205* sequence. KO of *OTX2-AS1* in D283 resulted in a significant reduction in cell viability (Fig. 2c), while OE of *OTX2-AS1* in ONS76 substantially increased cell viability (Fig. 2d).

To study whether *OTX2-AS1* may modulate the migration of medulloblastoma cells, we performed real-time cell analyses and continuously monitored cell migration in the different *OTX2-AS1* engineered cell models. KO-OTX2-AS1 D283 showed lower migration rates compared to the corresponding control cells (Fig. 2e). In contrast, OE-OTX2-AS1 ONS76 showed increased cell migration relative to control cells (Fig. 2f).

To investigate the potential oncogenic effects of *OTX2-AS1 in vivo*, we engrafted D283 into mice. Animals orthotopically injected with *OTX2-AS1* expressing D283 (Ctrl) showed significantly shorter survival compared to mice engrafted with corresponding KO-OTX2-AS1 D283 (Fig. 2g). Importantly, the deletion of *OTX2-AS1* in D283 and the OE of *OTX2-AS1* in ONS76 did not impact the expression of the adjacent *OTX2* (Fig. 2h), suggesting that the biological effect of *OTX2-AS1* was not merely mediated through an off-target effect or a direct regulatory feedback loop involving OTX2.

### B-cell lymphoma 2 (BCL-2) inhibitors specifically target medulloblastoma cells with high *OTX2-AS1* expression

**Fig. 3:**
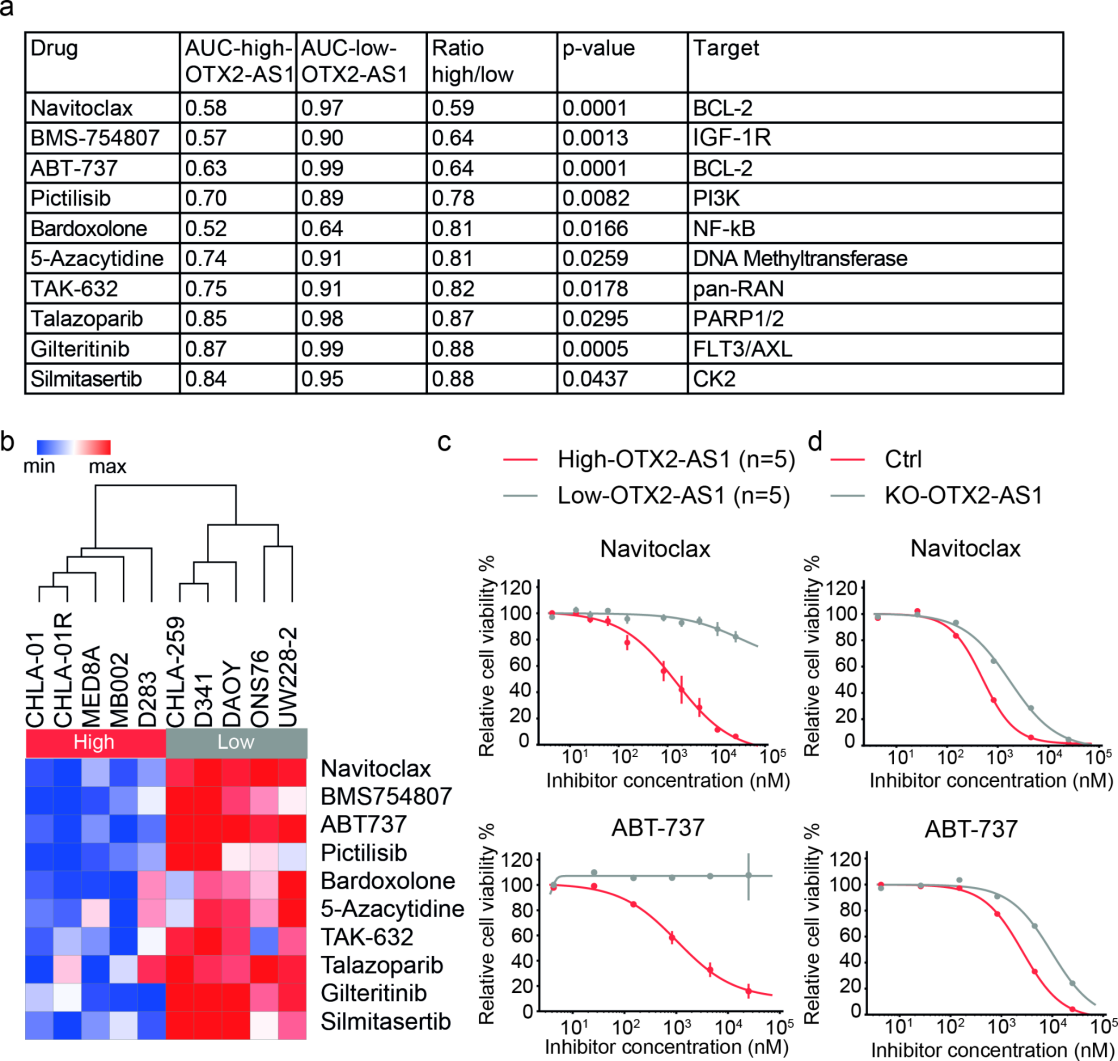
High-throughput drug screening of 579 clinically approved drugs identified ABT-737 and navitoclax as drug candidates active in medulloblastoma cells with high *OTX2-AS1* expression. **(a)** Ten drugs were more effective in medulloblastoma cell lines with high *OTX2-AS1* expression (D283, MB002, CHLA-01R, CHAL-01, MED8A) as compared to medulloblastoma cell lines with low *OTX2-AS1* expression (D341, CHLA-259, DAOY, UW228-2, ONS76). The AUC values were calculated from the modeled logistic 4-parameter dose-response curves. **(b)** Hierarchical clustering indicates two distinct segregations of medulloblastoma cell lines. The two identified clusters are correlated to the expression of *OTX2-AS1*. **(c, d)** Fitted dose-response curves show that medulloblastoma cells with high *OTX2-AS1* expression respond better to Navitoclax and ABT-737 than medulloblastoma cells with low-*OTX2-AS1* expression

To identify compounds that inhibit the survival of medulloblastoma cells in relation to their *OTX2-AS1* expression level, we performed high-throughput drug screening of 10 medulloblastoma cell lines. Of 579 tested drugs, 39 showed significant differences (*p* < 0.05) in response between cell lines with high and low *OTX2-AS1* expression. We identified the top 10 drugs that specifically targeted medulloblastoma cells with high *OTX2-AS1* cells based on the fold change (Fig. 3a). The corresponding heatmap (Fig. 3b) presents a clear separation of drug response in medulloblastoma cells according to high versus low *OTX2-AS1* expression. Two of the top three compounds with the lowest p-value are specific BCL-2 inhibitors, including ABT-737 and its orally available structural analog, navitoclax [34]. Dose-response curves indicated that the AUC of ABT-737 and navitoclax were significantly lower in medulloblastoma cells with high *OTX2-AS1* expression (Fig. 3c). To further confirm our results, we tested the drug response using KO-OTX2-AS1 D283 and its corresponding control. Both BCL-2 inhibitors were found to more effectively target high *OTX2-AS1* expressing control cells (Fig. 3d).

Furthermore, to assess any unwanted cytotoxic effects, we used human fibroblast cells (NHDF-Ad-1, NDHF-Ad-3, and NHDF-Ad-5) as non-neoplastic controls. The dose-response curves revealed that ABT-737 and Navitoclax did not impact the viability of these non-neoplastic cells (Fig. S2). Our results thus suggest that activation of the cell death machinery by pharmacological inhibition of BCL-2 could be a promising approach in medulloblastoma cells with high *OTX2-AS1* expression.

### Proteomic analysis showed increased essential mitochondrial proteins after KO of *OTX2-AS1*

**Fig. 4:**
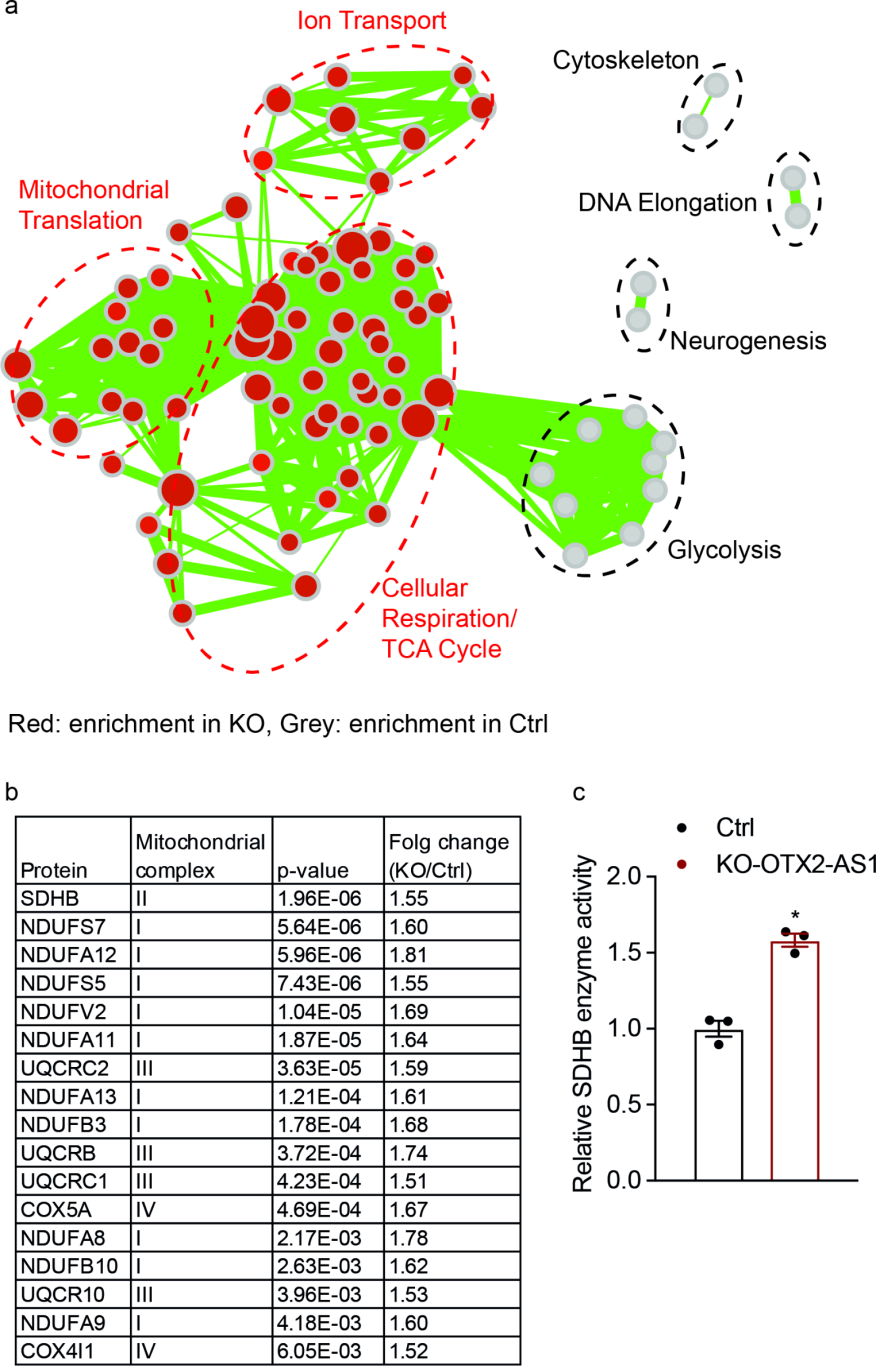
Proteomic and metabolomic analysis demonstrated increased SDHB enzyme activity in D283 medulloblastoma cells with KO of *OTX2-AS1*. **(a)** Identification of proteins from KO-OTX2-AS1 D283 or control cells using mass spectrometry. Gene Set Enrichment Analysis (GSEAhttp://software.broadinstitute.org/gsea/index.jsp) was performed using the C5 Gene Ontology biological process genes sets (MSigDb-http://software.broadinstitute.org/gsea/msigdb/index.jsp). The results were visualized using Cytoscape (www.cytoscape.org). The color of the nodes indicates the enrichment of KO (red) or Ctrl (grey), while the size of the nodes and the connecting lines represent the number of genes present either in the node or connecting the nodes, respectively. **(b)** The mitochondrial complex subunits were significantly upregulated after the KO of *OTX2-AS1*. The *p*-values were determined by the Bonferroni test ANOVA. **(c)** The enzyme activity of SDHB was calculated by determining the ratio of fumarate and succinate

To investigate the possible cellular effects of BCL-2 inhibitors on high *OTX2-AS1* expressing cells, we conducted proteomic analyses on the KO-OTX2-AS1 D283 and its corresponding control cells. After applying stringent cut-off criteria (*p* < 0.001), we identified 699 differentially expressed proteins, which were further analyzed based on gene ontology and protein function databases. These analyses revealed that depletion of *OTX2-AS1* resulted in increased expression of mitochondrial translation-related proteins and decreased expression of glycolysis-related proteins (Fig. 4a). In addition, we found significant upregulation of proteins essential for mitochondrial oxidative phosphorylation (OXPHOS) following KO of *OTX2-AS1*. These proteins included complex I subunits (NDUFA11, NDUFA12, NDUFA13, NDUFA8, NDUFA9, NDUFB10, NDUFB3, NDUFS5, NDUFS7, and NDUFV2) [35], a complex II subunit (succinate dehydrogenase b, SDHB) [36], complex III subunits (UQCR10, UQCRB, UQCRC1 and UQCRC2) [37], and complex IV subunits (COX5A, COX5B and COX4i1) [38] (Fig. 4b). We focused on the protein with lowest p-value, mitochondrial complex II enzyme SDHB, and measured its activity in control and KO cells. To this end, we measured the cellular amount of fumarate and succinate. In line with our initial proteomic data, we found that the fumarate/succinate ratio was increased in *OTX2-AS1* KO cells in relation to control cells, indicating enhanced SDHB enzyme activity (Fig. 4c).

## Discussion

LncRNAs play essential roles in developmental and differentiation processes, including neuronal development, as well as tumorigenesis [39]. Previous studies have reported on the roles of lncRNAs in the pathogenesis of medulloblastoma by interacting with various pathways, including the PI3K-AKT [21], MAPK [21], and p53 [40] signaling cascades. In this study, we provide molecular and functional evidence for a pro-tumorigenic role of increased expression of the lncRNA *OTX2-AS2* in medulloblastoma. By employing comparative methylation and transcriptome analyses, we observed high expression of *OTX2-AS1* in the WNT, G3, and G4 medulloblastoma groups but not in SHH medulloblastomas. This expression pattern parallels the expression pattern of *OTX2* in the different medulloblastoma groups, with immunohistochemical detection of OTX2 expression being considered a useful surrogate marker to separate the WNT, G3, and G4 medulloblastoma groups from the SHH medulloblastoma [41]. OTX2 is a member of the bicoid-like homeodomain (HD)-containing transcription factor family, which plays essential roles in embryo patterning, brain regionalization, and lineage specification [42]. *OTX2* amplification has been reported in subsets of G3 and G4 medulloblastomas [43, 44]. In addition, studies reported that *OTX2* expression enhanced medulloblastoma cell proliferation and tumorigenicity in vivo and that high *OTX2* expression levels are linked to shorter survival of medulloblastoma patients [45, 46].

*OTX2-AS1* maps adjacent to *OTX2* and is transcribed from the proximal promoter in the opposite direction of *OTX2* [31]. High *OTX2-AS1* expression has only been observed in the retina, while a low expression level was detected in the brain [31]. Here, we experimentally confirmed that *OTX2* and *OTX2-AS1* transcription is regulated from a bidirectional promoter and, hence, both transcripts are typically co-expressed in WNT, G3 and G4 medulloblastomas. In addition, we provide evidence that hypermethylation of the *OTX2/OTX2-AS1* promoter is common in SHH medulloblastoma and may thereby result in low expression levels of the *OTX2* and *OTX2-AS1* transcripts in this medulloblastoma group. However, this hypermethylation is not present in non-SHH medulloblastomas. Therefore, our findings indicate that the upregulation of *OTX2-AS1* expression in medulloblastoma is associated with an increase in *OTX2-AS1* copy number and the absence of *OTX2/OTX2-AS1* promoter methylation.

We demonstrated that *OTX2-AS1* promotes cell viability and migratory potential of medulloblastoma cells in vitro. Moreover, using an orthotopic xenograft mouse model of medulloblastoma, we observed longer survival in mice injected with *OTX2-AS1* KO D283 than *OTX2-AS1* expressing control cells. Our findings align with published data showing that aberrant *OTX2-AS1* expression is associated with poor survival in gastric cancer patients [47]. These observations suggest that compounds explicitly targeting tumor cells with elevated *OTX2-AS1* expression might constitute an innovative therapeutic approach. In this respect, we performed comparative high-throughput drug screening on medulloblastoma cells and identified two BCL-2 inhibitors (ABT-737 and Navitoclax) that specifically reduced cell viability of medulloblastoma cells with high *OTX2-AS1* expression. Furthermore, by using proteomics and metabolomics analyses, we identified and confirmed lower SDHB enzyme activity in high *OTX2-AS1* expressing cells. In acute myeloid leukemia, downregulation of SDHB sensitized leukemia cancer cells to BCL-2-inhibitor-induced apoptosis, which may explain the specific anti-tumor effect of the BCL-2 inhibitors towards high *OTX2-AS1* expressing cells in medulloblastoma [48].

BCL-2 inhibition has been shown to increase the response of cancer cells to radiation as well as chemotherapeutic agents [49]. Thus, combining BCL-2 inhibitors with standard therapies for medulloblastoma may achieve synergistic effects for improved therapeutic response. Unfortunately, both BCL-2 inhibitors identified in our drug screen show limited penetrance through the blood-brain barrier (BBB) [50]. However, delivery to brain tumors may be improved by, e.g., using nanocarrier-based “smart” drug delivery systems that facilitate drug transport across the BBB and enable sufficient intratumoral drug delivery [51]. BCL-2 is frequently overexpressed in medulloblastoma [52], and several studies focused on the growth-inhibitory effect of BCL-2 inhibition [53, 54]. Our findings add to these data by demonstrating the impact of *OTX2-AS1* expression on the response of cultured medulloblastoma cells to pharmacological BCL-2 inhibition.

In summary, our study provides molecular and functional evidence in support of the tumor-promoting role of *OTX2-AS1* in non-SHH medulloblastomas. In addition, *OTX2-AS1* expression may serve as a molecular biomarker to indicate the vulnerability of medulloblastoma cells toward pharmacological inhibition of BCL-2.

### Electronic supplementary material

Below is the link to the electronic supplementary material.

Supplementary Material 1
