# Supplementary material for: The long non-coding RNA OTX2-AS1 promotes tumor growth and predicts response to BCL-2 inhibition in medulloblastoma
Source: J Neurooncol. 2023 Nov 17;165(2):329–42. doi: 10.1007/s11060-023-04508-y (PMC10689561; doi:10.1007/s11060-023-04508-y)
Supplement: Supplementary file 1 — Supplementary Material 1 [file 11060_2023_4508_MOESM1_ESM.pdf]

Supplementary Information

The long non-coding RNA *OTX2-AS1* promotes tumor growth and predicts response to BCL-2 inhibition in medulloblastoma

Supplementary table legends

Table S1: Key resources including sources of cell lines and culture media.

Table S2: Concentrations of selection antibiotics.

Table S3: List of primer sequences used for qRT-PCR analyses.

F: Forward primer. R: reverse primer.

Table S4: Oligonucleotide primers used for plasmid construction.

Tables

Table S1: Key resources including sources of cell lines and culture media

Experimental Models	Source	Identifier	Culture media
UW228-3	Gift of Dr. Landgraf, University Hospital Duesseldorf (received: 2015)	RRID: CVCL_0573	Dulbecco's Modified Eagle Medium (DMEM, Thermo Fisher Scientific, Waltham, USA) supplemented with 10 % fetal bovine serum (FBS, Thermo Fischer Scientific)
ONS76	Gift of Dr. Landgraf, University Hospital Duesseldorf (received: 2015)	RRID: CVCL_1624	DMEM, 10 % FBS
D283	Gift of Dr. Landgraf, University Hospital Duesseldorf (received: 2015)	RRID: CVCL_1155	Eagle's Minimum Essential Medium (Thermo Fischer Scientific), 10% FBS
MED8A	Gift of Dr. Landgraf, University Hospital Duesseldorf (received: 2015)	RRID: CVCL_M137	DMEM, 10 % FBS
D425	Gift of Dr. Landgraf, University Hospital Duesseldorf (received: 2015)	RRID: CVCL_1275	DMEM, 10 % FBS
DAOY	Gift of Dr. Reifenberger, University Hospital Duesseldorf (received: 2015)	RRID: CVCL_1167	DMEM, 10 % FBS

CHLA-01	ATCC (received: 2015)	RRID: CVCL_B044	DMEM:F12 medium (Thermo Fisher Scientific), 20 ng/mL human recombinant EGF (Thermo Fisher Scientific), 20 ng/mL human recombinant FGF (Thermo Fisher Scientific), 2% (v/v) B-27 Supplement (Invitrogen, Waltham, USA)
CHLA-01R	ATCC (received: 2015)	RRID: CVCL_N534	DMEM:F12 medium (Thermo Fisher Scientific), 20 ng/mL human recombinant EGF (Thermo Fisher Scientific), 20 ng/mL human recombinant FGF (Thermo Fisher Scientific), 2% (v/v) B-27 Supplement (Invitrogen, Waltham, USA)
CHLA-259	Children's Oncology Group (received: 2015)	RRID: CVCL_M148	Iscove's Modified Dulbecco's Medium supplemented with 20% FBS, 1% Glutamax and 1X ITS (5000 ng/mL isulin, 5000 ng/mL transferrin, 5 ng/mL selenous acid)
MB002	Gift of Dr. Siddhartha, Stanford University (received: 2015)	RRID: CVCL_VU79	1:1 DMEM F12 (Thermo Fisher Scientific) and Neuralbasal-A Medium (Thermo Fischer Scientific), 1% Glutamax, 1 mM Sodium pyruvate (Thermo Fisher Scientific), 0.025 mM HEPES (Thermo Fisher Scientific), 1 % MEM NEAA (Thermo Fisher Scientific), 2 % B-27, 10 ng/mL EGF, FGF and LIF, 0.4 mg/mL Heparin
HEK293T	ATCC (received: 2015)	RRID: CVCL_0063	DMEM supplemented with 10 % FBS
NHDF-Ad (NHDF-Ad1, NHDF-Ad3, NHDF-Ad5)	Lonza (Basel, Switzland)		FGM-2 Growth Media (Cat# CC-3132, Lonza)

Table S2: Concentrations of selection antibiotics

	Puromycin (µg/ml)	Blasticidin (µg/ml)
ONS76	-	10
D283	0.5	-
MED8A	1	-

Table S3: List of primer sequences used for qRT-PCR analyses.

F: Forward primer. R: reverse primer.

**Genes**	**Sequence of qRT-PCR primers**	**Product size (bp)**	**Binding efficiency**
GAPDH	F: 5´-GTCAGCCGCATCTTCTTTTG-3´<br>R: 5´-GCGCCCAATACGACCAAATC-3´	100	95%
PGK1	F: 5´-GACAGCAGCCTTAATCCTCTG-3´<br>R: 5´-CTAACAAGCTGACGCTGGA-3´	122	93%
OTX2-AS1	F: 5´-TGAAAGCGATGATGATGTCG-3´<br>R: 5´-GCAACAAGAGCCAGGTAAGG-3´	155	95%
OTX2	F: 5´-AAGCACTGTTTGCCAAGACC-3´<br>R: 5´-TGACCTCCATTCTGCTGTTG-3´	154	80%

Table S4: Oligonucleotide primers for plasmid construction

	**Sequence of relevant primers**
gOTX2-AS1-1	F: 5´- caccGATAAAGAGCATGTTCTTAC-3´<br>R: 5´-aaacGTAAGAACATGCTCTTTATC-3´
gOTX2-AS1-2	F: 5´- caccgAATTGCAGACTGCCCGGCTC-3´<br>R: 5´-aaacGAGCCGGGCAGTCTGCAATT-3´
gOTX2-A1-Promoter-1	F: 5´-caccGAATTACGGGAGTGCGCACG-3´<br>R: 5´-aaacCGTGCGCACTCCCGTAATTC-3´
gOTX2-A1-Promoter-2	F: 5´-caccgCGTTATGACCCTCGTGGCGC-3´<br>R: 5´-aaacGCGCCACGAGGGTCATAACG-3´

Puro1	F: 5´-GTTGCAAAATTGCTCTTCAGCTAGCGCCACCATGACCGAGTAC-3´
Puro2	R: 5´-GGTTTGTGGGGACTGTACCTGCAGGAAAAAACCTCCCACACCTCCCCCTG-3´
OTX2-AS1-Donor1	F: 5´-GTTCCCAGGAGTCAAGAGTTTCTAG-3´
OTX2-AS1-Donor2	R: 5´-GTGCGCTTGGTCAGAGTCATGATGC-3´
OTX2-AS1-Donor3	F: 5´-CAACCGCAATGCAGGGAGTGAGAG-3´
OTX2-AS1-Donor4	R: 5´-CACATCTCGCACTTGTCACACAATC-3´

Supplementary figure legends:

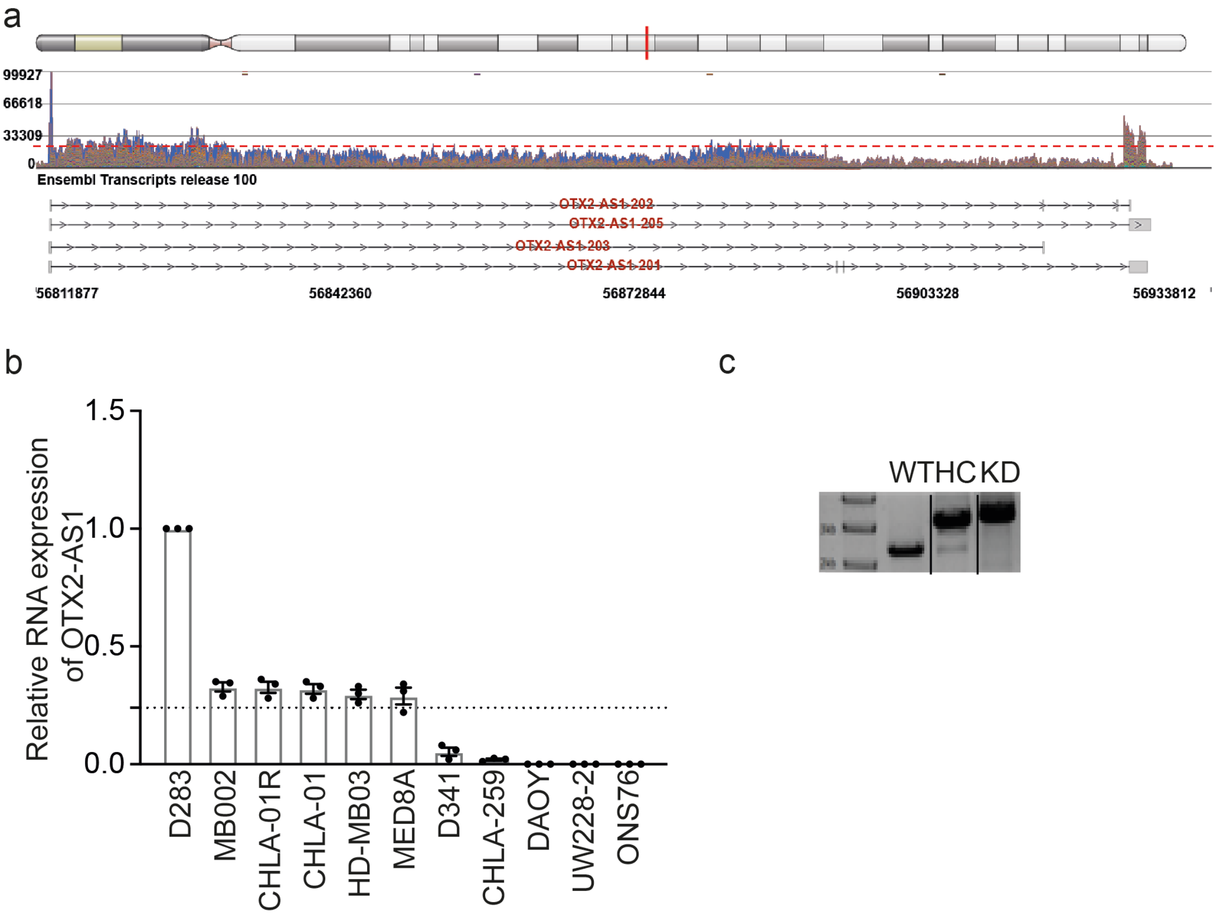

Fig .S1: *OTX2-AS1-205* expression in medulloblastoma and in different medulloblastoma cell lines. (a) RNAseq data from 41 flash-frozen primary medulloblastoma [26] show the *OTX2-AS1-205* transcript variant in medulloblastoma. (b) Measurement of *OTX2-AS1-205* expression in medulloblastoma cell lines using qRT-PCR. *GAPDH* and *PGK1* transcript levels were used as housekeeping controls. (c) Gel picture of electrophoresis showing PCR products from CRISPR/Cas9-targeted region in D283 medulloblastoma cells. Displayed are the ladder, wildtype D283 (WT), heterozygous construct (HC) and homozygote KO of *OTX2-AS1*.

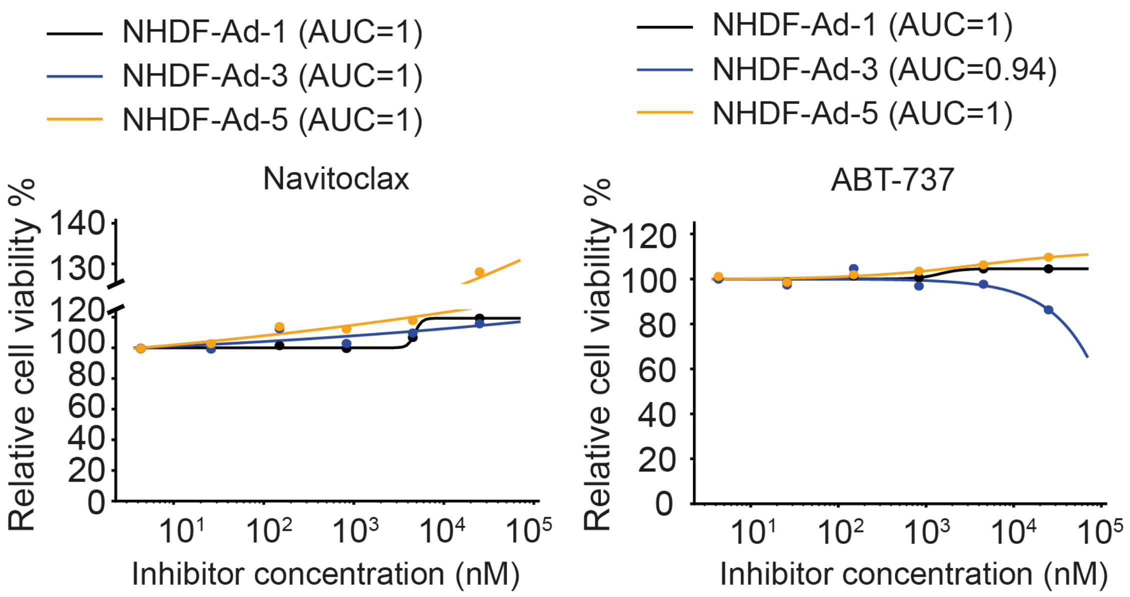

Fig. S2: Fitted dose-response curves demonstrate that navitoclax and ABT-7373 show limited toxicity in non-neoplastic human dermal fibroblasts.
